# Inhibition of mitotic kinase Aurora suppresses Akt-1 activation and induces apoptotic cell death in all-trans retinoid acid-resistant acute promyelocytic leukemia cells

**DOI:** 10.1186/1479-5876-9-74

**Published:** 2011-05-21

**Authors:** Duo-Rong Xu, Shan Huang, Zi-Jie Long, Jia-Jie Chen, Zheng-Zhi Zou, Juan Li, Dong-Jun Lin, Quentin Liu

**Affiliations:** 1 State Key Laboratory of Oncology in South China, Cancer Center, Sun Yat-sen University, 651 Dongfeng Road East, Guangzhou 510060, China; 2 Department of Hematology, First Affiliated Hospital, Sun Yat-sen University, 58 Zhongshan II Road, Guangzhou 510080, China; 3 Department of Hematology, Third Affiliated Hospital, Sun Yat-sen University, 600 Tianhe Road, Guangzhou 510630, China; 4 Sun Yat-sen Institute of Hematology, Sun Yat-sen University, 600 Tianhe Road, Guangzhou 510630, China

## Abstract

**Background:**

Aurora kinase ensures accurate chromosome segregation during cell cycle, maintaining genetic integrity in cell division. VX-680, a small-molecule Aurora kinase inhibitor, interferes with mitotic entry and formation of bipolar spindles. Here, we evaluated VX-680 as a potential agent for treatment of all-trans retinoid acid (ATRA)-resistant acute promyelocytic leukemia (APL) *in vitro*.

**Methods:**

CD11b expression was utilized to assess cell differentiation by flow cytometry. Immunofluorescence staining was conducted to analyze formation of cell monopolar spindle. Cell proliferation was evaluated by MTT assay. Sub-G1 population and Annexin V/PI staining were used to measure cell apoptosis. Hoechst 33342 staining was applied for identifying morphological changes in nucleus of apoptotic cell. Aurora-A (Aur-A) activation and the signaling pathways involved in apoptosis were detected by Western blot. JC-1 probe was employed to measure mitochondrial depolarization.

**Results:**

VX-680 inhibited Aur-A by reducing autophosphorylation at the activation site, Thr288, accompanied by producing monopolar mitotic spindles in APL cell line NB4-R2 that was resistant to ATRA. In addition, we found that VX-680 inhibited cell proliferation as assessed by MTT assay. Flow cytometry showed that VX-680 led to apoptotic cell death in both dose- and time-dependent manners by either Sub-G1 or Annexin V/PI analysis. Hoechst 33342 staining represented typical apoptotic cells with nuclear fragmentation in VX-680 treated cells. Importantly, VX-680 inhibition of Aurora kinase suppressed Akt-1 activation and induced mitochondrial depolarization, which eventually resulted in apoptosis by activation of caspase pathway, as indicated by increasing proteolytic cleavage of procaspase-3 and poly ADP ribose polymerase (PARP) in NB4-R2 cells.

**Conclusions:**

Our study suggested potential clinical use of mitotic Aurora kinase inhibitor in targeting ATRA-resistant leukemic cells.

## Background

Acute promyelocytic leukemia (APL), is characterized by t (15; 17) chromosomal translocation resulting in a fusion transcript of promyelocytic leukemia-retinoid acid receptor α (PML/RARα). PML/RARα represents a most curable subgroup of leukemia with the introduction of all-trans retinoid acid (ATRA) therapy [1,2]. ATRA binds to retinoic acid (RA) receptor, as a result of activating the target genes such as the myeloid-specific transcription factor C/EBP, thereby inducing differentiation of myeloid leukemia cells [3,4]. Although most APL patients respond to ATRA therapy, lack of effective treatment presents a serious challenge in non-ATRA responders.

Serine/threonine kinase Aurora family, including Aurora (Aur)-A, -B and -C, are playing important roles in chromosome segregation during cell cycle and genetic integrity in cell division [5,6]. Our previous study showed Aur-A was of importance for mitotic entry and formation of bipolar spindles [7]. Aur-A expression was aberrantly found in many solid tumors such as prostate, colon, pancreas, breast, and thyroid cancers [8-13]. Moreover, Aur-A expression level was correlated with prognosis and advanced clinical stage in head and neck squamous cell carcinoma [14,15]. Recently study showed that Aur-A kinase was highly expressed in acute myeloid leukemia (AML) patients and suppression of Aur-A induced AML cells apoptosis [16].

Recently, Aurora kinase small-molecule inhibitors have been considered as novel and potential anti-cancers agents. VX-680, showed anti-cancer activity in vivo in many solid cancers in preclinical experiment, and was demonstrated to inhibit multiple myeloma growth, especially in patients with RHAMM overexpression, and chronic myeloid leukemia (CML) with BCR-ABL mutations [17-19]. However, the potential usage of VX-680 inhibition of Aurora kinase in ATRA-resistant APL remains unknown.

Here we showed that Aurora kinase small-molecule inhibitor VX-680 led to mitotic defects in spindle and decreased expression of phosphorylated Aur-A at the activation site, Thr288 in APL cell line NB4-R2 that was resistant to ATRA. VX-680 induced apoptosis in NB4-R2 cells in both time- and dose-dependence. Importantly, we found that VX-680 down-regulated Akt-1 activation and induced mitochondrial depolarization, which resulted in caspase-3 associated apoptotic cell death. Thus, Aurora kinase inhibitor VX-680 offered as a novel therapeutic agent in treatment of ATRA-resistant APL patients.

## Materials and methods

### Reagents and Cells culture

VX-680 (Kava Tech, CA) was dissolved in dimethlsulfoxide (DMSO) to a stock concentration of 430 μM and stored at -20°C. Human APL NB4 and NB4-R2 cell lines, provided by Shanghai Institute of Hematology, Ruijin Hospital, were cultured in RPMI 1640 (Gibco) supplemented with 10% fetal bovine serum (FBS; Hyclone) at 37°C in a humidified 5% CO_2 _atmosphere.

### Cell differentiation assessment

To measure CD11b expression, NB4 and NB4-R2 cells (5 × 10^5^/ml) were plated in 6-well dishes and cultured with ATRA (1 μM). After 3 days, Cells were washed twice with PBS and incubated with primary mouse monoclonal CD11b antibody (Sigma) at 37°C for 1 hr. Then, the cells were washed once with PBS, and incubated with the secondary immunofluorescence antibody (FITC) for 1 hr in dark. Expression of CD11b on cell surface was measured by flow cytometry.

### Immunofluorescence staining

NB4-R2 cells were incubated with VX-680 at 2 nM for 24 hr. Cells were fixed in cold methanol for 20 min at 4°C and permeabilized in 0.5% TritonX-100 in PBS at room temperature (RT) for 15 min. Then cells were incubated with 1% BSA for 1 hr at RT to block nonspecific binding before the primary antibody reaction. Slides were incubated with the primary antibody to Aur-A, α-Tubulin at RT for 1 hr, followed by Alexa Flour 680 or FITC 488 conjugated antibody. After counterstained with DAPI (1 μg/ml), cells were visualized using a microscope (1000 ×, Olympus).

### Cell growth assay

Cell proliferation was assessed by MTT assay. NB4-R2 cells were plated in 96-well plates at 2.5 × 10^4 ^cells/ml in a final volume of 200 μl and exposed to different doses of VX-680 (0-10 nM) or ATRA. Sets of 5-wells were used for each dose. 20 μl of MTT solution (Sigma, 5 mg/ml) was added to each well at 24 hr and 48 hr. After cells were incubated at 37°C for another 4 hr, the medium was removed and 150 μl DMSO was added to solubilize the formazan. Finally, the absorbance (OD) was measured using a multiwell plate reader (Bio-Rad Microplate Reader).

### Sub-G1 population assay

NB4-R2 cells were collected and washed twice with PBS, then fixed by ice alcohol overnight at -20°C. Cells were then resuspended with PI at a concentration of 1.0 × 10^6 ^cells/ml. Quantification of Sub G1 population after PI staining was carried out using a FACS flow cytometer equipped with CellQuest software (BD).

### Measurement of apoptosis by Annexin V/PI analysis

After collecting and washing twice with PBS, VX-680 treated or untreated NB4-R2 cells were resuspended in the binding buffer (500 μl). FITC-Annexin-V (5 μl) was added to the cells followed by addition of 5 μl PI according to the protocol of the Annexin V-FITC/PI kit (EMD Biosciences). The samples were then incubated for 15 min in the dark at 4°C and subjected to flow cytometry evaluation.

### Identification and quantification of apoptotic cells with Hoechst 33342

Nuclear morphology of control and VX-680 treated cells was observed by staining cell nuclei with Hoechst 33342 (Sigma). Cells (at least 200 per slide) were incubated with Hoechst 33342 (10 μg/ml) for 15 min at RT and examined under a fluorescence microscope (Olympus) by using the MNU2 filter. Apoptotic cells were characterized by condensation of chromatin and/or nuclear fragmentation.

### Mitochondrial membrane potentials assay

JC-1 probe was employed to measure mitochondrial depolarization in NB4-R2 cells. Briefly, VX-680 treated cells were incubated with an equal volume of staining solution (5 μg/ml) at 37°C for 20 min and rinsed twice with PBS. Mitochondrial membrane potentials were monitored by determining the relative amounts of dual emissions from mitochondrial JC-1 by flow cytometry. Mitochondrial depolarization was indicated by an increase in the green fluorescence and a decrease in the red fluorescence intensity.

### Western blot analysis

NB4-R2 cells were lysed in RIPA buffer. The protein concentration was determined by Bradford method with BSA (Sigma) as the standard. Equal amounts of cell extract (40 μg) were subjected to electrophoresis in SDS-polyacrylamide gel and transferred to nitrocellulose membrane (Minipore). The membrane was blocked and then incubated with GAPDH (from Ambion), p-Aur-A/AIK (Thr288), cleaved PARP (Asp214), pAkt-1 (Ser473), cleaved caspase-3 (Asp175) and pGSK-3 (Ser9) antibodies (from Cell Signaling), at 4°C overnight, followed by incubation for 1 hr RT with appropriate secondary antibodies. Antibody binding was detected with an enhanced chemiluminescence kit and ECL film.

### Statistics

Statistical analysis was performed using SPSS version 11.0 (SPSS Inc.). The Student's t-test was used to make a statistical comparison between groups. The level of significance was set at p < 0.05.

## Results

### Aurora kinase small-molecule inhibitor VX-680 significantly suppresses the proliferation in a number of leukemic cell types

**Figure 1:**
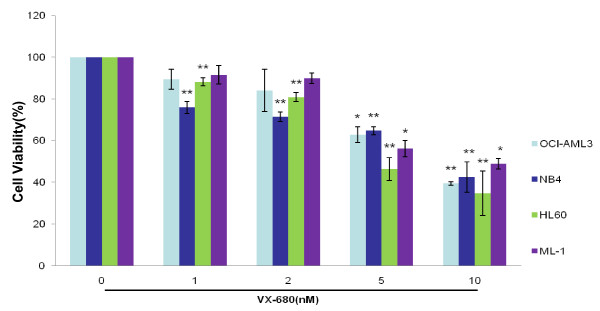
**VX-680 significantly suppresses the proliferation in a number of leukemic cell types**. OCI-AML3, NB4, HL-60 and ML-1 cells were incubated with increasing doses of VX-680 (1, 2, 5 and 10 nM) for 24 hr. Cell viability was measured by MTT assay. Data summarized three independent experiments, *p < 0.05, **p < 0.01, compared to control.

In order to demonstrate the specificity of Aurora inhibitory VX-680 on leukemia, OCI-AML3, NB4, HL-60 and ML-1 cells were treated with different doses of VX-680. As showed in Figure 1, VX-680 could inhibit cell growth rates in the 4 different leukemic cells we tested in a dose-dependent manner (ranging from 1 nM to 10 nM) after 24 hr treatment. However, VX-680 suppressed the proliferation in some solid tumor cell types with less potency, such as MCF-7 and Hela cancer cells (Figure S1, Additional file 1), suggesting that VX-680 was a potential anti-leukemic agent for various leukemic cell types.

### NB4-R2 cells are resistant to ATRA induced differentiation

**Figure 2:**
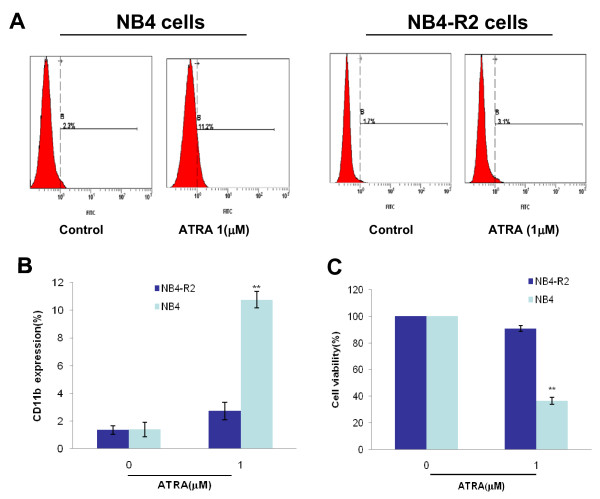
**NB4-R2 cells are resistant to ATRA induced differentiation**. (A) NB4 and NB4-R2 cells were treated with ATRA (1 μM) for 3 days, and CD11b-expressing population was measured by flow cytometry. (B) Data summarized three independent experiments, **p < 0.01, compared to control. (C) NB4 and NB4-R2 cells were treated with ATRA (1 μM), and the proliferation was measured by MTT assay. Data summarized three independent experiments, **p < 0.01, compared to control.

Promyeloid leukemic cell lines NB4 and NB4-R2 were treated with ATRA and cell differentiation was evaluated by quantifying CD11b expression, a marker of myeloid differentiation. After exposure of NB4 and NB4-R2 cells to ATRA (1 μM) for 72 hr, a mean of 10.76% NB4 cells were induced to express cell surface antigen CD11b. On contrast, only 1.4% of NB4-R2 cells expressed CD11b surface antigen (Figure 2A, B), confirming that NB4-R2 cells were resistant to ATRA-induced myeloid differentiation. MTT assay further showed that ATRA (1 μM) significantly inhibited NB4 cells growth, while the survival percentage was not statistically changed at this concentration in NB4-R2 cells (Figure 2C), indicating ATRA failed to inhibit NB4-R2 cells growth.

### VX-680 decreases pAur-A at the activation site and induces monopolar spindle in NB4-R2 cells

**Figure 3:**
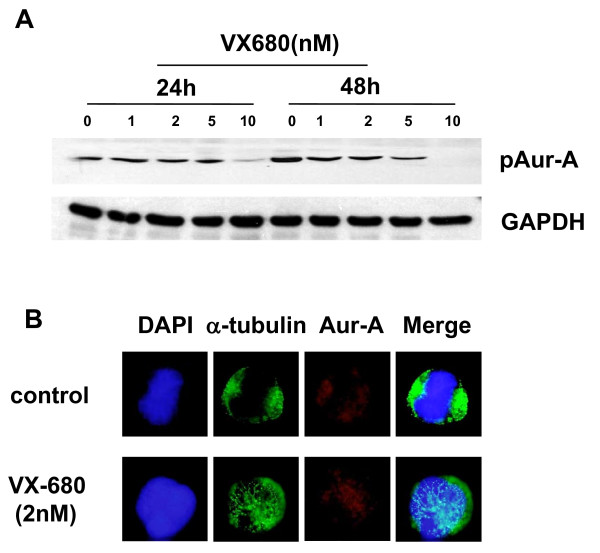
**VX-680 inhibits activation of Aur-A and induces monopolar spindle in NB4-R2 cells**. (A) VX-680 inhibited phosphorylation of Aur-A at Thr288 in NB4-R2 cell line. Cells were incubated with increasing doses of VX-680 for 24 hr and 48 hr and subjeceted to Western blot with antibodies of pAur-A and GAPDH. (B) The morphology of mitotic spindle was shown by immunofluorescence staining with anti-α-tubulin antibody and anti-Aur-A antibodies. Microtubules were stained as green, Aur-A protein as red, and nucleus as blue.

We studied the inhibition of Aurora kinases in NB4-R2 cells using VX-680. Aur-A activation was inhibited by VX-680 at different concentrations (1 nM, 2 nM, 5 nM, 10 nM) in a dose-dependent manner in NB4-R2 cells (Figure 3A). VX-680 (5 nM) significantly inhibited Aur-A by reducing autophosphorylation at the activation site, Thr288. Then, we examined the role of Aur-A inhibition by VX-680 in the formation of spindles. As assessed by immunofluorescence, control cells displayed normal bipolar spindles, presenting a clearly visible metaphase plate straddled by uniform radial arrays of microtubules from opposite poles (Figure 3B). In the contrast, VX-680 (2 nM) treated cells showed abnormal monopolar spindles, suggesting that the inhibition of Aurora kinase activity induced defects of mitotic spindle in VX-680 treated cells.

### VX-680 suppresses cell growth and induces cell apoptosis in NB4-R2 cells

**Figure 4:**
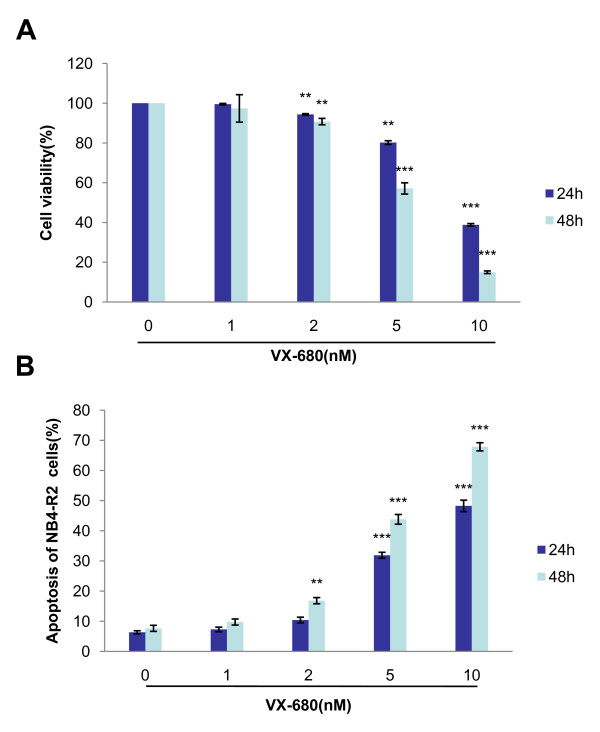
**VX-680 suppresses the proliferation of NB4-R2 cells and induces cell apoptosis**. NB4-R2 cells were incubated with increasing doses of VX-680 (1, 2, 5 and 10 nM) for 24 hr and 48 hr. (A) Cell viability was measured by MTT assay. (B) Sub-G1 population was detected by flow cytometry. Data summarized three independent experiments, **p < 0.01, ***p < 0.001, compared to control.

Next, we studied if VX-680 could suppress proliferation in NB4-R2 cells *in vitro*. NB4-R2 cells were treated with VX-680 at the concentration of 1 nM, 2 nM, 5 nM and 10 nM for 24 hr and 48 hr. Cell viability was assessed by MTT assay. At the concentration of 5 nM and 10 nM, VX-680 significantly inhibited the growth of NB4-R2 cells, with IC50 value of the anti-proliferation effect of VX-680 at 7.10 nM for 24 hr and 4.29 nM for 48 hr in NB4-R2 cells (Figure 4A).

**Figure 5:**
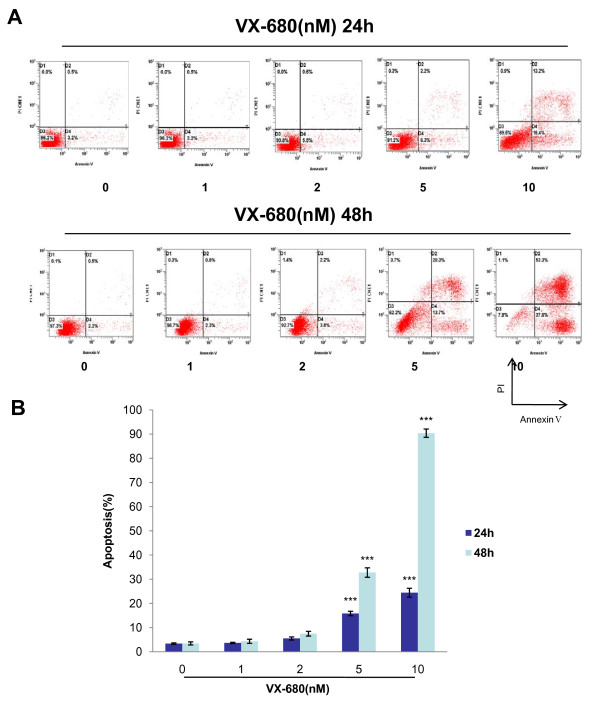
**VX-680 induces apoptosis of NB4-R2 cells by Annexin V/PI staining**. NB4-R2 cells were treated with VX-680 at different concentrations for 24 hr and 48 hr. (A) Apoptotic cells were measured by Annexin V/PI staining. (B) Data summarized three independent experiments, **p < 0.01, ***p < 0.001, compared to control.

**Figure 6:**
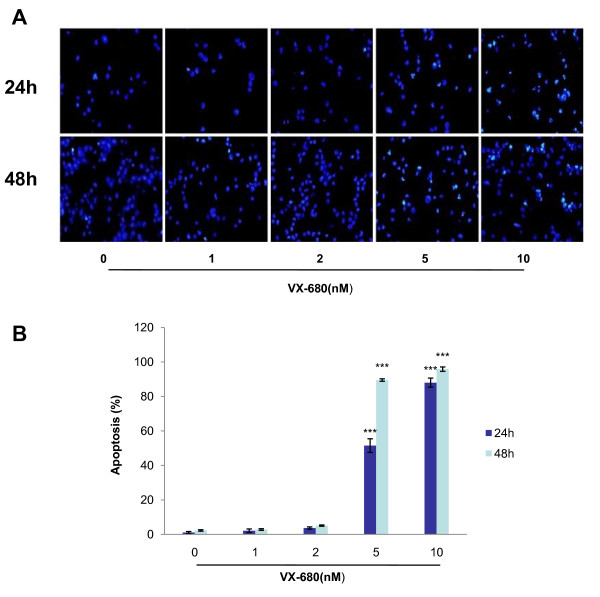
**Morphological changes in nucleus after induction of apoptosis by VX-680**. (A) VX-680 treated or untreated cells were stained with Hoechst 33342, and observed by fluorescence microscopy (magnification, 400×). (B) Data summarized three independent experiments, ***p < 0.001, compared to control.

We further assessed whether VX-680 could induce apoptosis in NB4-R2 cells. Incubation of VX-680 (1 nM, 2 nM, 5 nM and 10 nM) led to an increased apoptosis for 24 hr (7.3%, 10.45%, 31.9% and 48.27%, respectively) and 48 hr (9.77%, 16.83%, 43.8% and 67.85%, respectively) by assessing the sub-G1 population (Figure 4B). In addition, apoptotic cells were also detected by both Annexin V/PI staining and immunofluorescent staining with Hoechst 33342. Annexin V/PI staining showed that percentage of apoptosis were 3.66%, 5.52%, 15.83%, 24.43% respectively for 24 hr, and 4.35%, 7.47%, 32.77%, 90.4% respectively for 48 hr at the indicated doses of VX-680 (Figure 5). Similarly, control cells which were stained by Hoechst 33342 were uniformly blue in viable cells, whereas the apoptotic cells showed bright blue dots in the nuclei, representing the nuclear fragmentation, especially at VX-680 concentration of 5 nM and 10 nM (Figure 6). These results indicated that the apoptotic NB4-R2 cells were induced by Aurora kinase small-molecule inhibitor VX-680 in both dose- and time-dependent manners.

### VX-680 reduces mitochondrial membrane potentials and induces cellular caspase activation in NB4-R2 cells

**Figure 7:**
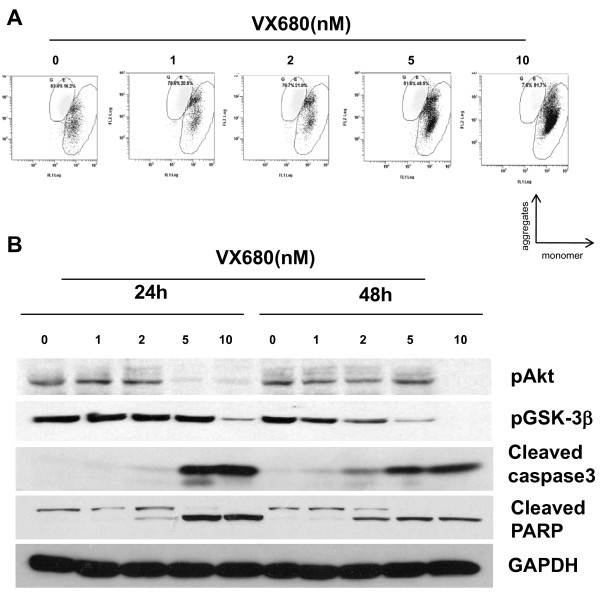
**VX-680 induces mitochondrial depolarization and cellular caspase activation in NB4-R2 cells**. (A) VX-680 treated NB4-R2 cells were stained with JC-1 probe and measured by flow cytometry. X- and Y-axes were indicative of monomer and aggregates, respectively. Data shown is a representative of three independent experiments. (B) NB4-R2 cells were collected, lysed and subjected to Western blot analysis with cleaved caspase-3, cleaved-PARP, pAkt-1 (Ser473), pGSK-3β (Ser9) specific antibodies. GAPDH was used as a loading control. Data shown is a representative of three independent experiments.

Further, we investigated the molecule events triggered by Aurora inhibition. Reduction of mitochondrial membrane potential is one of the molecule events for early apoptosis. Changes in mitochondrial membrane potential was assessed by monitoring JC-1, which accumulates in mitochondria forming red fluorescent aggregates at high membrane potential, whereas exits mainly in cytosol forming green fluorescent monomer, presenting a collapse of membrane. In our study, VX-680 treated cells showed loss of red fluorescence and production of obvious green fluorescence, suggesting reduction of mitochondrial membrane potentials. At different concentrations of VX-680 (1 nM, 2 nM, 5 nM and 10 nM), the percentage of NB4-R2 cells emitted green fluorescence was 20.9%, 21.8%, 48.5% and 91.7%, respectively, indicative of mitochondrial membrane depolarization in a dose-dependent manner. In comparison, control cells emitted mitochondrial red fluorescence with less green fluorescence (Figure 7A). Western blot analysis showed that inhibition of Aurora kinase with VX-680 for 24 hr and 48 hr induced amounts of cleaved caspase-3 expression. The cleavage of the PARP polymerase, a major target for caspases, was also detected in VX-680 treated cells. At dose of 5 nM, cleaved caspase-3 and PARP expression was dramatically increased in NB4-R2 cells (Figure 7B). Interestingly, VX-680-induced activation of caspase pathway was correlated with down-regulation of Akt-1 phosphorylation at the activation site, Ser473 and decreased the level of phosphorylated GSK-3β at Ser9, the downstream of Akt-1 (Figure 7B). Thus, VX-680 suppressed Akt-1 activation, reduced mitochondrial membrane potentials and induced NB4-R2 cells apoptosis by activation of caspase pathway.

## Discussion

Aurora kinases are important for the accurate execution of mitotic events. Aur-A played a significant role in ensuring the centrosome segregation and spindle assemble [20,21]. The expression of Aur-A were commonly increased in various malignant tumors [9,10]. Our recent work has showed that inhibition of Aur-A induced cell apoptotic death of laryngeal and oral squamous cell carcinoma as well as nasopharyngeal carcinoma [22-24]. In addition, Aur-A was overexpressed in bone marrow mononuclear cells (BMMCs) in a significant proportion of *de novo *AML patients [16]. Small-molecule Aurora kinase inhibitor VX-680 had anti-leukemic effect for various leukemic cell types and was considered to be a potential targeting agent (Figure 1). However, the role of VX-680 in treating ATRA-resistant APL cells has not been evaluated. In this study, we showed that NB4-R2 cells were resistant to ATRA by detecting expression of CD11b (Figure 2). VX-680 reduced the autophosphorylation of Aur-A at the activation site, Thr288 (Figure 3A) and caused formation of monopolar structures in NB4-R2 cells (Figure 3B). In both dose- and time-dependent manners, VX-680 suppressed NB4-R2 cells growth (Figure 4A) and induced cells apoptosis (Figure 4B, 5, and 6). Moreover, we observed VX-680 induced mitochondrial depolarization by flow cytometry (Figure 7A) and importantly, caspase pathway was activated, which was associated with down-regulation of Akt-1 phosphorylation at the activation site, Ser473 (Figure 7B). Our results suggest that VX-680 is a potential novel agent for APL treatment, and Aurora kinase may serve as a promising therapeutic target for ATRA-resistant APL patients.

APL is characterized by a balanced reciprocal translocation between chromosomes 15 and 17, which results in the fusion between PML gene and RARα. Since the introduction of ATRA in the treatment and optimization of the ATRA-based regimens, the complete response (CR) rate was raised up to 90%-95% and 5-year disease free survival (DFS) was to 74% [2,25-27]. However, resistance and relapse were still frequently observed in APL cases after treatment with ATRA. Alterations of the PML/RARα protein point mutation have been the major ATRA-resistant mechanism [28-30]. NB4-R2, is a ATRA-resistant subclone of the NB4 APL cell line, which changes the amino acid Gln903 to an in-phase stop codon, generating a truncated form of PML/RARα which has lost 52 amino acids at its C-terminal end [31]. In addition to the point mutation, fusions with PLZF in t(11;17)(q23;q21) expressed in APL cells may be other mechanisms of resistance to ATRA [32]. Therefore, it is urgent to identify novel agents against ATRA-resistant APL.

Recently, many clinical drugs have been used in the management of APL patients with ATRA-resistant, but were associated with some severe adverse effects [33]. Emerging kinase small molecule inhibitors were tested for potent anti-leukemic activity with less adverse effects. VX-680 was designed to target the ATP-binding site of the Aurora kinases, and was reported to be active in anticancer therapy with affinity for Aur-A (Ki = 0.6), B (Ki = 18), and C (Ki = 4.6) [34]. VX-680 also inhibited other protein kinases, including Flt-3 (Ki = 30) and MAPK (Ki > 1000), albeit with less potency. VX-680 reduced phosphorylation of Aur-A on its activation site Thr288, therefore suppressing phosphorylation of mitotic Histone H3 at Ser10, arresting cell cycle in G2/M phase and blocking proliferation in multiple tumor cell types [22-24,34]. In addition, VX-680 induced formation of monopolar spindles, a phenotype of inactive Aur-A mutant [35], which led to mitotic catastrophe and apoptosis in cancer cell lines. We and others have demonstrated additional mechanism of VX-680 inhibition of Aurora in suppressing Akt activation, down-regulating NF-κB activity, and subsequently reducing survival and migration in malignant cells [24,36,37].

In this report, we found that VX-680 inhibited Aurora kinase and presented anti-tumor activation in NB4-R2 cells, suggesting a possible novel and potent target in treating ATRA-resistant APL. Here, we clearly showed that VX-680 inhibited growth of NB4-R2 cells and induced cell apoptosis in vitro in the concentration of 1-10 nM. At the dose range, VX-680 inhibited Aur-A phosphorylation at Thr288. In addition, VX-680 destructed the bipolar spindle structure, a typical phenotype of Aurora suppression. Thus, our data demonstrated a potential role of an Aurora inhibitor VX-680 in ATRA-resistant APL targeted therapeutics.

Tumor cells apoptotic mechanism involves an interaction of a number of key cellular regulatory pathways, including cell proliferation pathway, cell survival pathway, caspase activation pathway, tumor suppressor pathway, death receptor pathway, mitochondrial pathway and protein kinase pathway. Most cells apoptosis pathway is through mitochondrial-mediated pathway, which is mostly regulated by Bcl-2 family, including the anti-apoptotic and pro-apoptotic factors, and subsequently induces cell apoptosis by controlling the release of cytochrome c from membrane of mitochondria [38]. In our study we found that VX-680 induced the mitochondrial depolarization and finally resulted in caspase pathway activation. Phosphatidylinositol 3-kinase (PI3K)/AKT signaling pathway plays crucial roles in cell growth, migration and invasion [24,37]. Akt is significant for regulating growth factor-stimulated cell survival response though its substrates proteins such as GSK-3, Bad and forkhead transcription factors [39]. It has been reported that high expression of Akt is relative with survival, proliferation of leukemic cells in AML and inhibition of activation of Akt can result in suppression of cell growth [40,41]. In the present study, phosphorylation of Akt-1 and GSK3β, the downstream of Akt-1, was decreased in VX-680 treated NB4-R2 cells. In addition, we also found that Akt signaling inhibitor API-2 could inhibit Akt-1 phosphorylation and induced apoptosis (data not show), indicating NB4-R2 cell apoptotic death induced by VX-680 might be due to down-regulation of Akt activation in NB4-R2 cells.

## Conclusions

Taken together, we showed that Aurora kinase-directed small-molecule inhibitor VX-680 suppressed cell growth, and induced apoptosis in NB4-R2 cells, offering an opportunity for a novel approach targeting Aurora signaling pathway in ATRA-resistant APL treatment.

## Abbreviations

ATRA: all-trans retinoid acid; APL: acute promyelocytic leukemia; Aur: Aurora; PARP: poly ADP ribose polymerase; PML/RARα: promyelocytic leukemia-retinoid acid receptor α; AML: acute myeloid leukemia; CML: chronic myeloid leukemia; DMSO: dimethlsulfoxide; NF-κB: nuclear factor-κB.

## Competing interests

The authors declare that they have no competing interests.

## Authors' contributions

DRX participated in analysis and interpretation of data, and critical revision of the manuscript. SH, ZJL, JJC and ZZZ have made substantial contributions to acquisition of data. JL and DJL participated in critical analysis of results. QL participated in conception and design, analysis and interpretation of data, and critical revision of the manuscript. All authors read and approved the final manuscript.

## Supplementary Material

Additional file 1 **Figure S1 - VX-680 does not effectively suppress the proliferation in MCF-7 and Hela cells**. MCF-7 and Hela cells were incubated with increasing doses of VX-680 (1, 2, 5 and 10 nM) for 24 hr. Cell viability was measured by MTT assay. Data summarized three independent experiments, *p < 0.05, compared to control. Click here for file
