# Supplementary figures and images for: Inhibition of mitotic kinase Aurora suppresses Akt-1 activation and induces apoptotic cell death in all-trans retinoid acid-resistant acute promyelocytic leukemia cells
Source: J Transl Med. 2011 May 21;9:74. doi: 10.1186/1479-5876-9-74 (PMC3224588; doi:10.1186/1479-5876-9-74)

## Slide 1
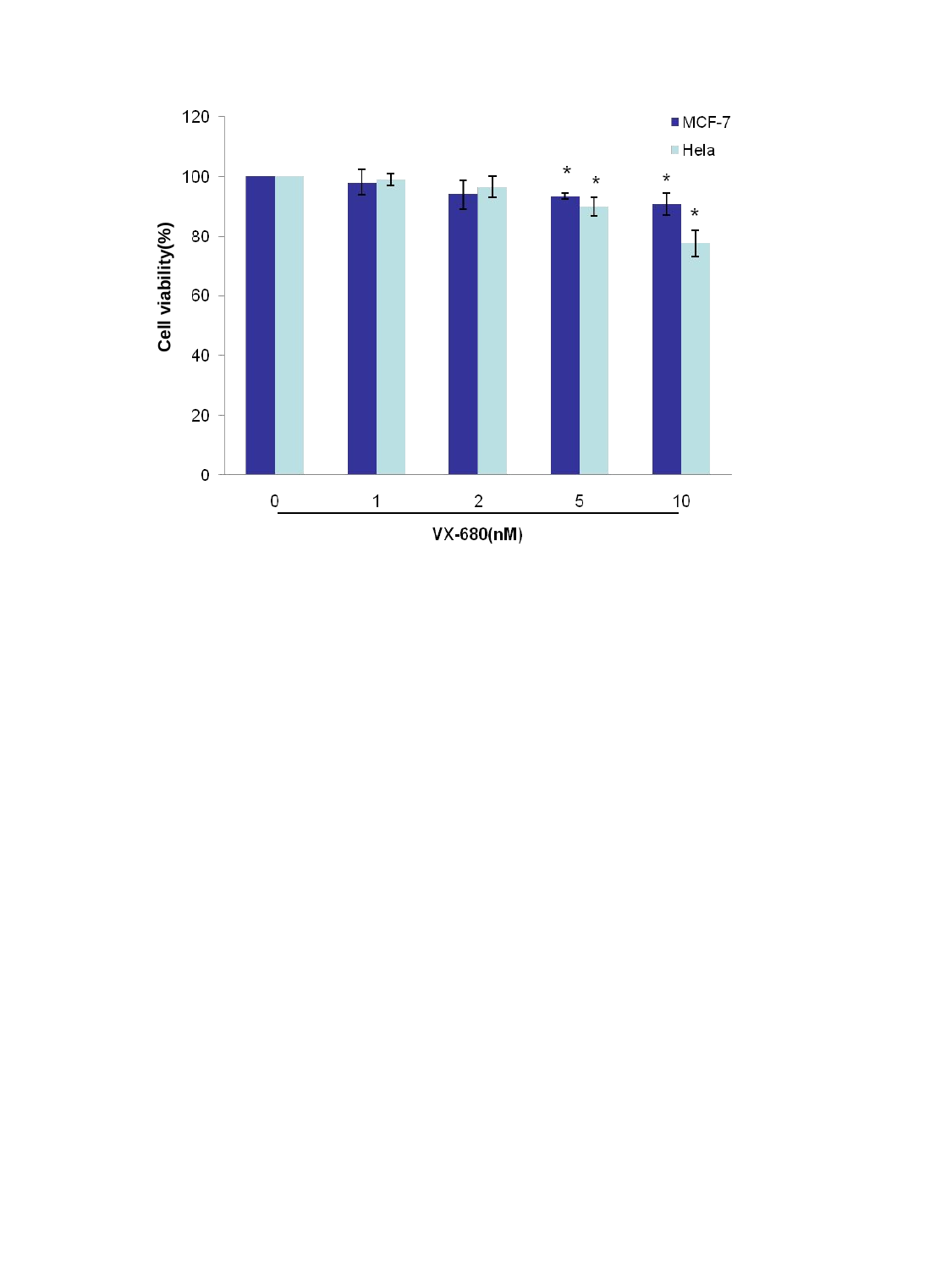

Cell viability(%)

Supplement: Additional file 1 — Figure S1 - VX-680 does not effectively suppress the proliferation in MCF-7 and Hela cells. MCF-7 and Hela cells were incubated with increasing doses of VX-680 (1, 2, 5 and 10 nM) for 24 hr. Cell viability was measured by MTT assay. Data summarized three independent experiments, *p < 0.05, compared to control. [file 1479-5876-9-74-S1.PPT]
